# Tuning Excited-State Properties in Pyrrolo[3,2-*b*]pyrrole-Based Donor–Acceptor Emitters via Molecular Conformation and Conjugation Control

**DOI:** 10.3390/molecules30214228

**Published:** 2025-10-29

**Authors:** Taotao Gan, Jie Su, Feiyang Li, Qiuxia Li, Chao Shi

**Affiliations:** School of Environmental and Chemical Engineering, Jiangsu University of Science and Technology, Zhenjiang 212100, China

**Keywords:** intramolecular charge transfer, local excitation, photoluminescence, donor–acceptor

## Abstract

Nitrogen-fused conjugated heterocycles have attracted growing interest owing to their tunable electronic properties and potential in organic optoelectronics. In this study, two centrosymmetric donor–acceptor-type emitters PP-6F and PPA-3F were designed by incorporating trifluorophenyl and anthracene acceptor units into a pyrrolo[3,2-*b*]pyrrole (PP) framework. The experimental and theoretical results reveal that subtle modulations in molecular conformation and π-conjugation pathways strongly affect the excited-state characteristics. PP-6F, featuring a nearly coplanar donor–acceptor configuration, exhibits efficient π-electron delocalization and a dominant local excitation (LE) emission with a large oscillator strength. In contrast, the bulky anthracene in PPA-3F increases the donor–acceptor dihedral angle, reduces conjugation coupling, and promotes orbital separation, leading to a hybrid intramolecular charge transfer and local excitation (ICT/LE) excited state. The rigid anthracene framework suppresses structural reorganization and nonradiative decay, allowing PPA-3F to retain a relatively high oscillator strength despite its charge-transfer nature. This work demonstrates that fine-tuning donor–acceptor dihedral angles and conjugation continuity within PP-based systems is an effective strategy for balancing LE and ICT emissions and developing high-efficiency nitrogen-fused organic emitters and scintillators.

## 1. Introduction

Conjugated heteroaromatic compounds composed of two or more aromatic rings have attracted considerable attention in organic electronics such as organic solar cells and organic light emitting diodes (OLEDs) owing to their unique electron rich characteristics and efficient π electron delocalization [1,2,3,4]. The extensive delocalization of intramolecular π electrons effectively narrows the energy gap between the highest occupied molecular orbital (HOMO) and the lowest unoccupied molecular orbital (LUMO), enabling efficient absorption of visible and even near-infrared light [5,6]. Moreover, long range π conjugation and a fused ring backbone contribute to enhanced charge carrier mobility [7]. Among various heteroaromatic systems, nitrogen containing fused ring compounds stand out due to their higher electronegativity, deeper frontier energy levels, and superior hole transporting properties [8]. Their molecular frameworks are readily amenable to chemical modification and solution processing, which make them highly promising for advanced organic optoelectronic devices such as OLEDs and organic field effect transistors [9].

The electronic density of nitrogen heterocycles can be flexibly tuned through structural design and connection modes, allowing them to exhibit a wide range of electronic properties from strong electron donors to effective electron acceptors. Among them, pyrrolo[3,2-*b*]pyrrole (PP) derivatives, characterized by highly electron rich fused five membered rings, have been widely employed as donor cores [10]. By introducing electron withdrawing groups as acceptors, donor–acceptor-type molecules can be constructed to achieve tunable optoelectronic properties [11,12,13]. Although such systems often show significantly red shifted absorption and emission spectra, the enhanced intramolecular charge transfer (ICT) transition usually reduces the oscillator strength of the excited state [13,14,15,16]. Furthermore, charge transfer delocalization and the spatial separation of the HOMO and LUMO orbitals can effectively decrease the energy gap (Δ*E*_ST_) between the singlet excited state (S_1_) and the triplet excited state (T_1_) [17]. A smaller Δ*E*_ST_ facilitates the reverse intersystem crossing (RISC) process in thermally activated delayed fluorescence (TADF), thereby improving the utilization efficiency of triplet excitons. Since radiative transitions from triplet states are spin forbidden in traditional pure organic molecules, enhancing triplet utilization efficiency is crucial for the performance of OLEDs and X-ray scintillation materials [18,19,20,21,22]. However, because of the strong electron donating ability and rigid fused structure of the PP core, introducing strong electron withdrawing substituents may induce excessive molecular planarity between donor and acceptor units. This promotes uniform π electron delocalization across the entire molecule and reduces the ICT character, leading to local excitation (LE). Therefore, precise control of the dihedral angle between donor and acceptor fragments is essential to regulate electronic transition behavior and consequently modulate photophysical properties of PP-based systems.

**Scheme 1:**
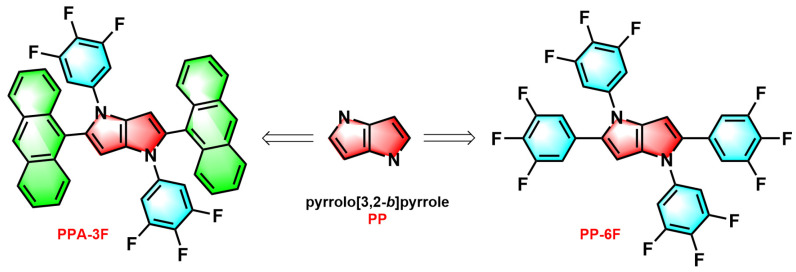
The molecular structures of PPA-3F and PP-6F.

Guided by these considerations, two centrosymmetric luminescent compounds PPA-3F and PP-6F (Scheme 1) were successfully synthesized by incorporating a trifluorophenyl unit and a bulky anthracene unit, respectively, as electron withdrawing moieties into the pyrrolo[3,2-*b*]pyrrole framework [18]. Experimental results revealed distinctly different radiative behaviors in their excited states. PP-6F mainly exhibits efficient LE emission, while PPA-3F displays characteristic charge transfer emission. A comprehensive investigation combining theoretical calculations and single crystal X-ray diffraction (SCXRD) analysis was conducted to elucidate the microscopic origin of these differences. The results show that the larger steric hindrance of the anthracene unit in PPA-3F increases the dihedral angle between the anthracene and the PP core, thereby enhancing the ICT character. Detailed analysis of bond lengths and molecular conformations further demonstrates how steric effects and conjugation topology govern the excited state transition behavior of donor-acceptor systems. Finally, the scintillation performance of these compounds under X-ray excitation was investigated, highlighting their potential application in radiation detection.

## 2. Results and Discussion

### 2.1. Synthesis

The target compounds PPA-3F and PP-6F were synthesized through a one pot tandem reaction combining a Debus Radziszewski condensation with a subsequent Knoevenagel condensation [15]. Specifically, the corresponding aromatic amines and aldehydes were reacted under mildly acidic conditions in the presence of Fe(OTs)_3_ (Iron(III) *p*-toluenesulfonate) as a catalyst, affording the desired products in yields of 15% and 11%, respectively. The molecular structures of both compounds were confirmed by comprehensive characterization including proton nuclear magnetic resonance (^1^H NMR), carbon nuclear magnetic resonance (^13^C NMR), and mass spectrometry. The structures were further unambiguously identified by single crystal X-ray diffraction (SCXRD) analysis.

### 2.2. Photophysical Properties

**Figure 1:**
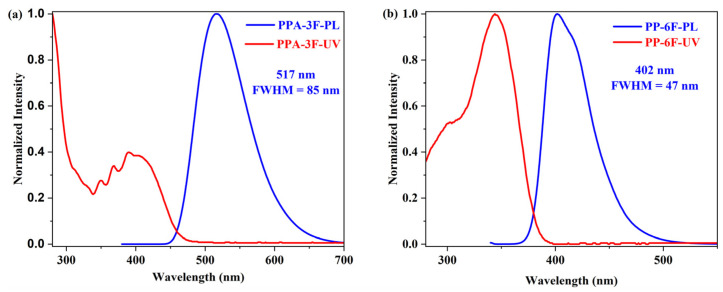
UV-Vis absorption (red line) and photoluminescence (blue line) spectra of the compounds (**a**) PPA-3F and (**b**) PP-6F in CH_2_Cl_2_. For PPA-3F, λₑₓ = 370 nm; for PP-6F, λₑₓ = 300 nm.

The photophysical behavior of compounds PP-6F and PPA-3F was first investigated in dichloromethane solution by ultraviolet visible absorption (UV-Vis) and photoluminescence (PL) spectroscopy (Figure 1 and Figure S1). For PPA-3F, the absorption spectrum exhibits two main regions. The absorption band between 340 and 400 nm is attributed to the π→π* transition of the anthracene unit, while the lower energy band ranging from 400 to 450 nm can be clearly assigned to the intramolecular charge transfer (ICT) transition from the PP donor to the anthracene acceptor. In contrast, PP-6F shows a strong absorption band between 320 and 400 nm, which is mainly attributed to the LE transition between the PP core and the trifluorophenyl unit.

**Figure 2:**
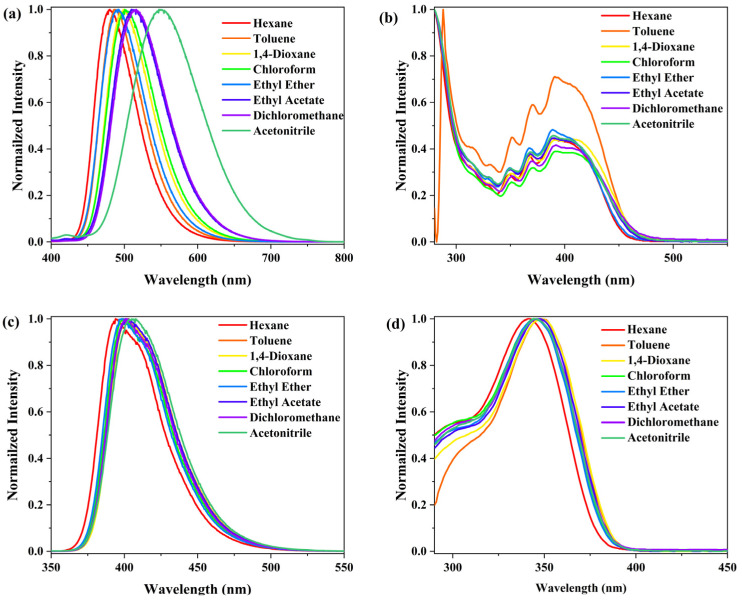
Emission (**a**,**c**) and absorption (**b**,**d**) spectra of PPA-3F (**a**,**b**) and PP-6F (**c**,**d**) measured in solvents of varying polarity. For PPA-3F, λₑₓ = 370 nm; for PP-6F, λₑₓ = 300 nm.

In the emission spectra, PPA-3F exhibits a broad and red shifted emission band centered at 517 nm with a full width at half maximum (FWHM) of 85 nm (3094 cm^−1^) and a large Stokes shift of 110 nm (5343 cm^−1^), which is a typical feature of an ICT excited state. PP-6F, on the other hand, displays a much narrower and symmetric emission profile centered at 402 nm with a FWHM of 47 nm (2826 cm^−1^) and a Stokes shift of 56 nm (4169 cm^−1^) indicating a dominant LE character. Compared with the unmodified PP derivatives PP-8 and PP-9 (Scheme S1) [23], the introduction of multiple trifluorophenyl electron-withdrawing groups leads to slight blue shifts in both absorption and emission spectra, with shifts of only about 6 nm. This indicates that the incorporation of fluorine substituents alone does not significantly affect the electronic transition characteristics of the unmodified PP compounds. To further confirm the nature of their excited states, solvent dependent UV-Vis and PL spectra (Figure 2 and Figure S2) were recorded in solvents with different polarities. The emission maximum of PP-6F shows only a slight red shift of 13 nm and its absorption peak remains nearly unchanged with a shift less than 7 nm. In sharp contrast, PPA-3F shows a pronounced positive solvatochromic effect, where the emission maximum red shifts from 480 nm in nonpolar solvents to 550 nm in polar solvents, with a total shift of 70 nm. This strong solvent dependence provides compelling evidence for a typical ICT excited state in PPA-3F.

**Figure 3:**
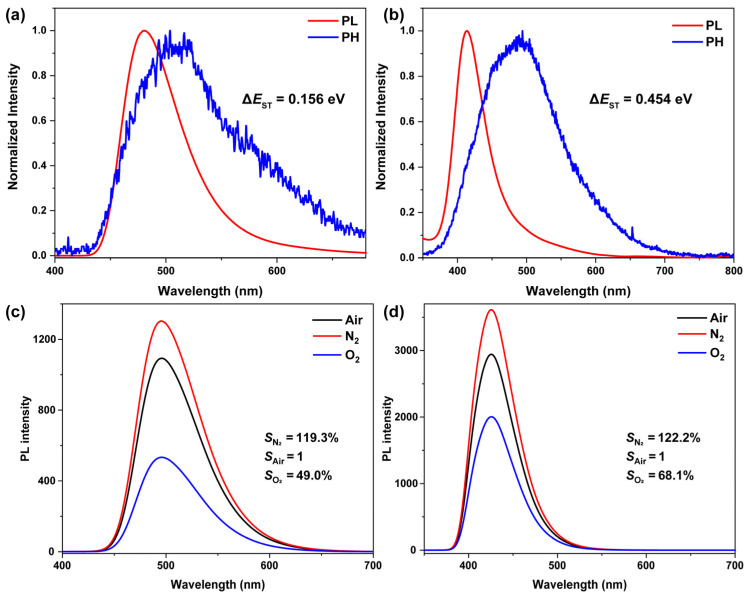
Steady-state photoluminescence (red line) and phosphorescence (blue line) spectra of compounds (**a**) PPA-3F (λ_ex_ = 370 nm) and (**b**) PP-6F (λ_ex_ = 340 nm) in toluene at 77 K. Steady-state emission spectra of compounds (**c**) PPA-3F and (**d**) PP-6F in toluene under air, nitrogen, and oxygen atmospheres (λ_ex_ = 300 nm).

To evaluate the potential participation of triplet excitons in the emission process, the PL spectra (Figure 3 and Figure S3) of both compounds were measured at 298 K and 77 K. The phosphorescence peaks of PPA-3F and PP-6F were observed at 512 and 491 nm, respectively. Based on the energy difference between fluorescence and phosphorescence maxima, the Δ*E*_ST_ were estimated to be 0.156 eV for PPA-3F and 0.454 eV for PP-6F. The smaller Δ*E*_ST_ of PPA-3F favors the involvement of triplet excitons through the RISC process. Considering that dissolved oxygen is a known quencher of triplet excitons, the PL intensities (Figure 3c,d) were further monitored under nitrogen and oxygen atmospheres to probe triplet participation at room temperature. Upon oxygen introduction, the emission intensities of both compounds decreased significantly, confirming their oxygen sensitivity and the possible involvement of triplet states. Taking the emission intensity in air as a reference, PPA-3F showed a reduction of 51.0% upon oxygen bubbling and a 19.3% enhancement after nitrogen purging, whereas PP-6F exhibited only a 31.9% reduction and a 22.2% enhancement. The higher oxygen sensitivity of PPA-3F correlates well with its smaller Δ*E*_ST_, suggesting a more efficient RISC process and a stronger contribution from triplet excitons that are more susceptible to quenching by oxygen. The decay curves of the compounds in dichloromethane were measured at room temperature. The nanosecond-scale decay profiles indicate that the emissions originate from fluorescence (Figure S4). No microsecond-scale decay component was observed at room temperature, confirming the absence of phosphorescence emission under these conditions.

**Figure 4:**
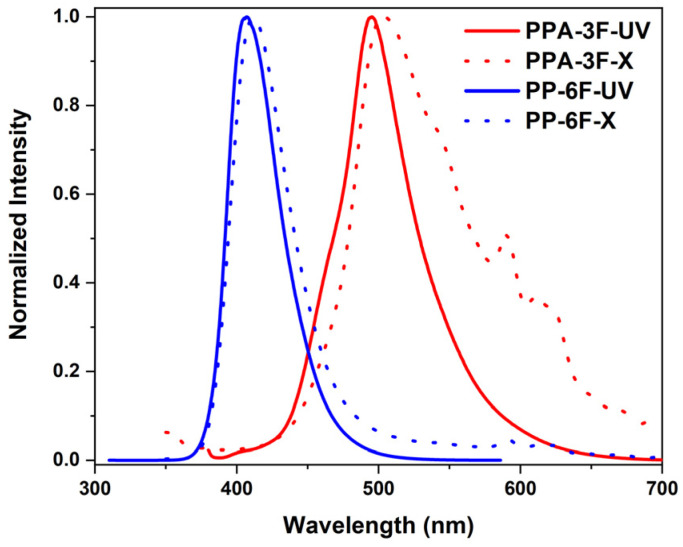
Emission spectra of PPA-3F (red lines) and PP-6F (blue lines) under ultraviolet excitation (solid lines) and X-ray excitation (dashed lines). For PPA-3F, λₑₓ = 370 nm; for PP-6F, λₑₓ = 300 nm.

Although the ICT transition facilitates a smaller Δ*E*_ST_, it can also result in decreased emission intensity due to reduced spatial overlap between the occupied and unoccupied molecular orbitals. To evaluate their solid-state emission performance relevant to practical applications, both compounds were doped into polystyrene (PS) films at a concentration of 1 wt%, and their PL spectra (Figure 4 and Figure S5) were recorded. In PS films, the emission peaks of PPA-3F and PP-6F were located at 496 and 406 nm with FWHMs of 62 and 43 nm, respectively. Notably, PP-6F exhibited a much stronger emission intensity (Figure S6) than PPA-3F. In the powder state, PP-6F exhibits distinct vibronic emission peaks (Figure S7). In contrast, PPA-3F shows composite emission behavior. The emission peak at 500 nm can be attributed to monomolecular emission, as its position is close to that observed in solution and in PS films, while the emission around 550 nm is likely associated with aggregated species in the powder state. This observation further supports our conclusion that the LE dominated character of PP-6F, associated with its smaller dihedral angle and efficient conjugation, ensures a higher oscillator strength and emission efficiency. In contrast, the enhanced ICT nature of PPA-3F arising from its larger dihedral angle and greater orbital separation favors the RISC process but sacrifices intrinsic emission intensity. These findings highlight the critical importance of achieving a balance between molecular conformation and electronic transition type in the design of high-performance luminescent materials. Considering the capability of both compounds to utilize triplet excitons, we further investigated their radioluminescence (RL) spectra under X-ray excitation (Figure 4). The emission peaks under X-ray irradiation are largely consistent with those observed under UV-excitation, suggesting that the primary radiative transition originates from the S_1_ state. Notably, the FWHM of PPA-3F RL is broadened, which may result from an increased population of triplet excitons generated under X-ray irradiation, enhancing the phosphorescence contribution via direct T_1_ emission. The X-ray radioluminescence demonstrated by these compounds underscores their significant potential as the organic scintillators.

### 2.3. Single Crystal Structure Analysis

**Figure 5:**
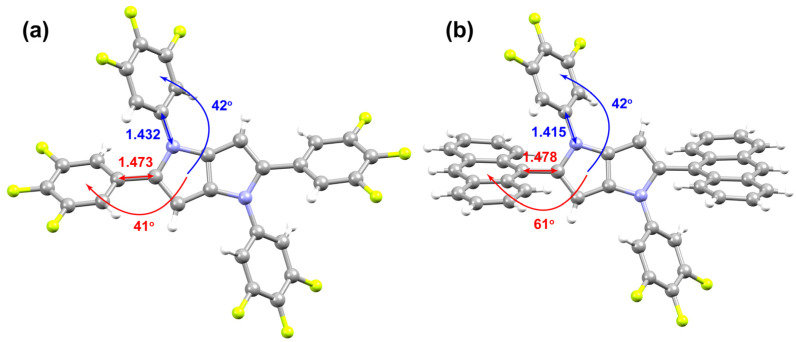
Bond lengths and dihedral angles in the single-crystal structures of (**a**) PP-6F and (**b**) PPA-3F.

The pronounced differences in the photoluminescence properties between PP-6F and PPA-3F can be attributed to variations in their molecular conformations and electronic delocalization pathways as revealed by single crystal analysis. In PP-6F, the dihedral angles between the two electron-withdrawing trifluorophenyl groups and the electron rich PP core are 41° and 42° (Figure 5), respectively. This nearly coplanar geometry ensures efficient and continuous π electron delocalization, leading to an excited state dominated by LE characteristics. In contrast, although the dihedral angle between the *N*-linked trifluorophenyl and PP units in PPA-3F remains around 42°, the much bulkier anthracene moiety introduces substantial steric hindrance, increasing the dihedral angle between the anthracene and PP units to 61°. This large torsion strongly suppresses π orbital overlaps between the anthracene and PP fragments, thereby promoting effective separation between the occupied and unoccupied molecular orbitals and favoring the transformation of the excited state from an LE to an ICT character. Such conformational twisting is recognized as a key factor responsible for the distinct emission behaviors of these two molecules.

Bond length parameters further support the structural interpretation. The C–C bond connecting the anthracene and PP units in PPA-3F is 1.478 Å (Figure 5), slightly longer than the corresponding bond (1.473 Å) between the *C*-linked trifluorophenyl and PP units in PP-6F, indicating weaker π conjugation in PPA-3F. In addition, the C–N bond lengths between the *N*-linked trifluorophenyl and PP units are 1.415 Å for PP-6F and 1.432 Å for PPA-3F. The elongation of the C–N bond in PPA-3F suggests a reduction in the n–π conjugation between the PP and the *N*-linked trifluorophenyl fragment. Moreover, in PP-6F, the *C*-linked trifluorophenyl substituent on the PP framework facilitates continuous π delocalization across the molecular skeleton, forming an overall planar conjugated structure. This extended conjugation reduces the interaction between the nitrogen lone pair and the *N*-linked trifluorophenyl ring, leading to a partial loss of double bond character in the C–N linkage and a slight increase in its bond length.

### 2.4. Theoretical Calculations

**Figure 6:**
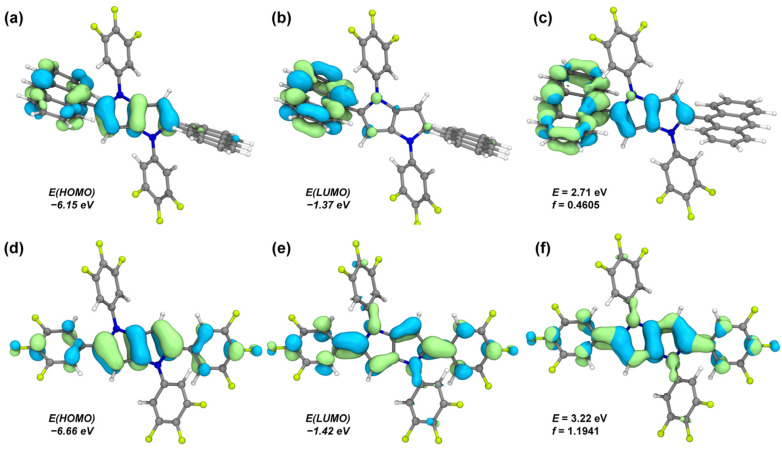
Molecular orbitals based on the S_1_ geometries and the corresponding hole–electron distributions for the S_0_ → S_1_ excitation of compounds (**a**–**c**) PPA-3F and (**d**–**f**) PP-6F. In the hole–electron distribution maps (**c**,**f**), the blue and green regions represent the hole and electron distributions, respectively.

Based on the experimentally determined crystal structures, density functional theory (DFT) and time dependent DFT (TD-DFT) calculations were performed to gain deeper insight into the excited state characteristics of the two compounds. Considering that electronic excitation typically induces geometric relaxation and symmetry reduction toward the minimum of the S_1_ potential surface, all excited state optimizations were carried out without any symmetry constraints. Figure 3 displays the frontier molecular orbitals calculated at the optimized S_1_ geometries. The HOMO and LUMO energy levels (Figure 6) are calculated to be −6.15 and −1.37 eV for PPA-3F, and −6.66 and −1.42 eV for PP-6F, respectively. The smaller energy gap of PPA-3F compared with PP-6F is consistent with its red shifted absorption and emission spectra observed experimentally. In PPA-3F, the HOMO is mainly distributed over the PP donor core and the anthracene moiety, while the LUMO is localized primarily on the anthracene acceptor. This spatial separation clearly supports its ICT nature. By contrast, both the HOMO and LUMO of PP-6F are extensively delocalized over the PP unit and the *C*-linked trifluorophenyl rings. Such broad delocalization leads to a lower HOMO energy level and a larger HOMO-LUMO energy gap, in agreement with its blue shifted emission spectrum.

**Figure 7:**
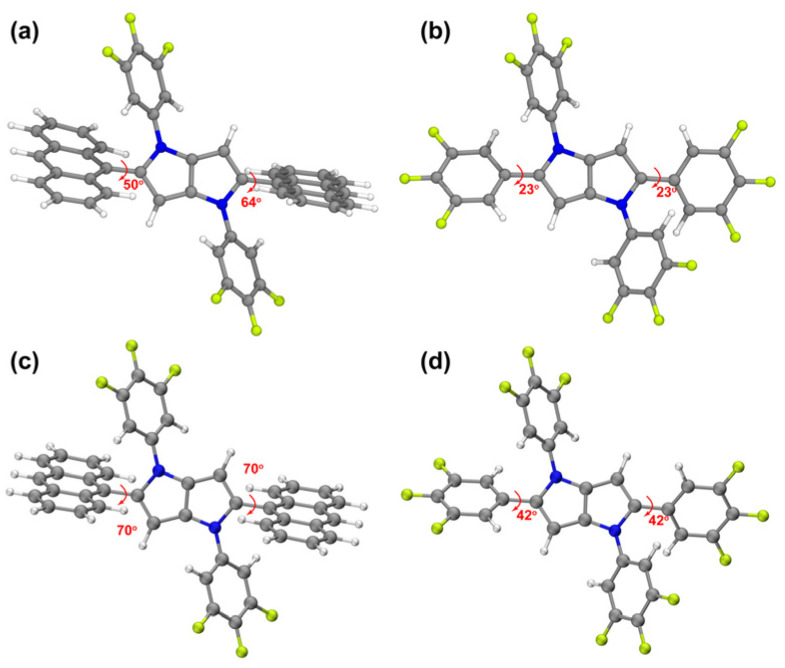
Dihedral angles in the S_1_ (**a**,**b**) and S_0_ (**c**,**d**) optimized geometries of compounds (**a**,**c**) PPA-3F and (**b**,**d**) PP-6F.

The optimized S_1_ geometries further confirm the conformational modulation of conjugation. In the excited state, the dihedral angle between the *C*-linked trifluorophenyl and PP units in PP-6F is reduced to 23° (Figure 7), whereas that between the anthracene and PP units in PPA-3F remains as large as 50°. This result provides strong evidence that the *C*-linked trifluorophenyl configuration in PP-6F promotes more efficient π conjugation and a planar backbone. The calculated S_1_ excitation energies and oscillator strengths are 3.22 eV and 0.4605 for PPA-3F, and 2.71 eV and 1.1941 for PP-6F, respectively. The S_1_ and T_1_ excited-state energies (Figure S8) were calculated based on the optimized geometry of the S_1_ state to obtain more reasonable energy gap values. The T_1_ energy is much lower than S_1_, and the energy gaps between S_1_ and the adjacent triplet states are 0.184 eV and 0.393 eV, which are in good agreement with the experimental values. Due to the large energy gap between the T_1_ state and the higher-lying triplet states, the experimentally observed phosphorescence may originate from anti-Kasha emission of a higher triplet state.

Analysis of the electron–hole distributions for the S_0_→S_1_ transitions reveals distinct excitation characteristics [24]. In PP-6F, both the hole and electron densities (Figure 6f) are highly overlapped and delocalized across the *C*-linked trifluorophenyl and PP fragments, confirming a predominantly LE transition nature, consistent with its larger oscillator strength and the experimentally observed intense PL emission. In contrast, PPA-3F exhibits a significant charge transfer from the PP donor unit to the anthracene acceptor (Figure 6c). Notably, the HOMO of PPA-3F still shows partial electron density on the anthracene ring, indicating a mixed ICT and LE excited state character. This hybrid nature allows PPA-3F to retain a large oscillator strength while possessing strong charge-transfer characteristics, offering an advantage over conventional D-A systems that exhibit purely ICT transitions.

**Figure 8:**
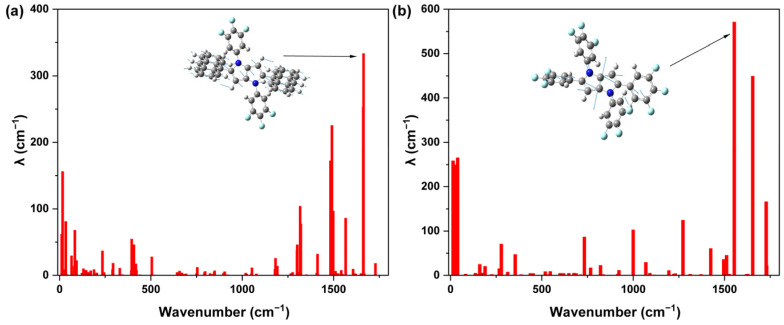
Calculated reorganization energies versus the normal mode wavenumber for the S_1_ → S_0_ emission process of (**a**) PPA-3F and (**b**) PP-6F.

To gain deeper insight into the origin of the higher oscillator strength in PPA-3F, we analyzed the reorganization energy (λ_re_) associated with the S_1_→S_0_ emission process using vibrational mode decomposition (Figure 8). The results reveal that PPA-3F possesses a markedly lower total reorganization energy (16.84 kcal/mol) than PP-6F (19.11 kcal/mol). This counterintuitive trend, where the LE dominated PP-6F has a higher λ_re_, can be attributed to their distinct excited-state relaxation behaviors. Although PP-6F exhibits mainly LE-type emission, the electrons are extensively delocalized across the PP core and two C-linked trifluorophenyl groups, forming a large π-conjugated framework that undergoes substantial planarization upon excitation. This structural relaxation induces significant bond-length alternation within the skeleton, with the 1553 cm^−1^ stretching mode of the PP core contributing most strongly to the reorganization energy. In contrast, PPA-3F features a mixed ICT/LE excited state and a more rigid molecular structure. The inherent rigidity of the anthracene unit and the smaller change in the dihedral angle between the PP and anthracene moieties upon excitation effectively suppress large-scale geometrical reorganization. This leads to a low reorganization energy, where the major vibrational contribution arises from the 1664 cm^−1^ stretching mode involving the PP and anthracene moieties. Consequently, the hybrid excited-state character and enhanced structural rigidity of PPA-3F jointly minimize the S_1_ to S_0_ geometric distortion, thereby reducing nonradiative decay and supporting its higher oscillator strength.

## 3. Conclusions

In summary, two centrosymmetric donor–acceptor-type emitters, PP-6F and PPA-3F, were successfully constructed by introducing trifluorophenyl and anthracene acceptor units into the pyrrolo[3,2-*b*]pyrrole (PP) framework. Comprehensive experimental investigations combined with theoretical calculations reveal that subtle modulations in molecular conformation and conjugation pathways exert a profound influence on the excited-state properties and emission behaviors of these compounds. Benefiting from its nearly coplanar molecular structure, PP-6F enables efficient delocalization of π-electrons between the PP core and the *C*-linked trifluorophenyl groups, leading to a predominantly LE state with a large oscillator strength and high emission efficiency. In contrast, the incorporation of the bulky anthracene moiety in PPA-3F significantly increases the dihedral angle between donor and acceptor fragments, weakens π-conjugation coupling, and promotes spatial separation of frontier orbitals, thereby endowing the molecule with pronounced hybrid ICT/LE characteristics. The theoretical calculations analysis confirms that this hybrid character and the inherent rigidity of the anthracene core effectively suppress structural reorganization, enabling PPA-3F to retain a relatively high oscillator strength despite its strong charge-transfer character.

This study highlights an effective molecular design strategy based on the synergistic regulation of molecular conformation and electronic structure, providing valuable guidance for the development of high-efficiency nitrogen-fused polycyclic emitters and potential scintillation materials with tunable luminescent properties.

## 4. Materials and Methods

### 4.1. Synthetic Details

All chemicals and reagents used in this work were obtained from Energy Chemical (Shanghai, China) and were used without further purification unless otherwise stated.

2,5-di(anthracen-9-yl)-1,4-bis(3,4,5-trifluorophenyl)-1,4-dihydropyrrolo[3,2-*b*]pyrrole (PPA-3F): 3,4,5-trifluoroaniline (0.82 g, 5.6 mmol, 1.0 equiv) and 9-anthracenecarboxaldehyde (1.15 g, 5.6 mmol, 1.0 equiv) were weighed into a dry two-neck round-bottom flask. Acetic acid (4 mL) and toluene (4 mL) were added, and the mixture was heated and stirred at 50 °C for 1 h. Iron(III) *p*-toluenesulfonate hexahydrate (0.23 g, 0.336 mmol, 0.06 equiv) was then introduced, followed by the slow addition of 2,3-butanedione (0.24 g, 2.8 mmol, 0.5 equiv). The reaction mixture was stirred overnight at 50 °C. After the reaction was complete, the mixture was cooled to room temperature. The resulting solid was collected by vacuum filtration and washed with acetic acid. The filter cake was dried to afford the target product as a yellow solid (0.313 g, 15%). ^1^H NMR (400 MHz, CDCl_3_) δ 8.55 (s, 2H), 8.08–7.99 (m, 8H), 7.50–7.44 (m, 8H), 6.73–6.67 (m, 4H), 6.64 (s, 2H). ^13^C NMR (100 MHz, CDCl_3_) δ 132.25, 131.24, 131.00, 129.53, 128.76, 128.68, 126.61, 126.48, 126.06, 125.40, 107.66, 98.97. EI-MS (*m*/*z*): 718.8 (M^+^, 100%). The carbon signals coupled with fluorine atoms are relatively weak.

1,2,4,5-tetrakis(3,4,5-trifluorophenyl)-1,4-dihydropyrrolo[3,2-*b*]pyrrole (PP-6F): 3,4,5-trifluoroaniline (0.82 g, 5.6 mmol, 1.0 equiv) and 3,4,5-trifluorobenzaldehyde (0.89 g, 5.6 mmol, 1.0 equiv) were weighed into a dry two-neck round-bottom flask. Acetic acid (6 mL) and toluene (6 mL) were added, and the mixture was heated and stirred at 50 °C for 1 h. Iron(III) *p*-toluenesulfonate hexahydrate (0.23 g, 0.336 mmol, 0.06 equiv.) was then introduced, followed by the slow addition of 2,3-butanedione (0.24 g, 2.8 mmol, 0.5 equiv). The reaction mixture was stirred overnight at 50 °C. After the reaction was complete, the mixture was cooled to room temperature. The resulting solid was collected by vacuum filtration and washed with acetic acid. The filter cake was dried to afford the target product as a white solid (0.193 g, 11%).^1^H NMR (400 MHz, CDCl_3_) δ 6.91–6.85 (m, 4H), 6.83–6.77 (m, 4H), 6.37 (s, 2H). ^13^C NMR (100 MHz, CDCl_3_) δ 152.85, 150.10, 134.27, 133.81, 131.70, 128.17, 112.13, 109.74, 96.59. EI-MS (*m*/*z*): 626.25 (M^+^, 100%). The carbon signals coupled with fluorine atoms are relatively weak.

### 4.2. Measurements

^1^H NMR and ^13^C NMR spectra were recorded on a Jeol JNM-ECZ400S/L1 NMR System Spectrometer (Tokyo, Japan), and the molecular weight was characterized by the Agilent Technologies 5973N (EI) (Santa Clara, CA, USA). The UV-vis absorption spectra were recorded on the Hitachi U-3900 UV-Visible Spectrophotometer (Tokyo, Japan). Photoluminescence spectra were measured on the HITACHI fluorescence spectrophotometer F-4700 and Edinburgh FS5 (Livingston, UK). The photoluminescence decay curves were measured using an Edinburgh FS5. The phosphorescence spectra were recorded using the phosphorescence mode of the F-4700 spectrometer with a chopping speed of 40 Hz. For compound PP-6F, the emission bands at 414 nm and 488 nm are assigned to fluorescence and phosphorescence at 77 K, respectively. For compound PPA-3F, the emission bands at 481 nm and 512 nm correspond to fluorescence and phosphorescence, respectively. Accordingly, the calculated Δ*E*_ST_ values for the two compounds are 0.454 eV and 0.156 eV, respectively.

### 4.3. Crystal

Single crystals of PPA-3F and PP-6F were grown from mixed solutions of dichloromethane and ethanol by the solvent diffusion method. CCDC 2494807–2494808 contains the supplementary crystallographic data for this paper.

Crystal Data for PPA-3F. CCDC number: 2494807. C_46_H_24_F_6_N_2_ (*M* =718.67 g/mol): monoclinic, space group P2_1_/n (no. 14), *a* = 8.732(4) Å, *b* = 14.019(5) Å, *c* = 13.610(6) Å, *β* = 97.103(7)°, *V* = 1653.2(11) Å^3^, *Z* = 2, *T* = 293.15 K, μ(MoKα) = 0.108 mm^−1^, *Dcalc* = 1.444 g/cm^3^, 14,852 reflections measured (5.526° ≤ 2Θ ≤ 56.658°), 4121 unique (*R*_int_ = 0.0373, R_sigma_ = 0.0395) which were used in all calculations. The final *R*_1_ was 0.0468 (I > 2σ(I)) and *wR*_2_ was 0.1179 (all data).

Crystal Data for PP-6F. CCDC number: 2494808. C_30_H_10_F_12_N_2_ (*M* =626.40 g/mol): triclinic, space group P-1 (no. 2), *a* = 11.4197(9) Å, *b* = 11.7546(10) Å, *c* = 11.8294(9) Å, *α* = 111.681(4)°, *β* = 99.552(4)°, *γ* = 102.922(4)°, *V* = 1383.0(2) Å^3^, *Z* = 2, *T* = 302.0 K, μ(CuKα) = 1.305 mm^−1^, *Dcalc* = 1.504 g/cm^3^, 15,308 reflections measured (8.26° ≤ 2Θ ≤ 136.732°), 5014 unique (*R*_int_ = 0.0497, R_sigma_ = 0.0621) which were used in all calculations. The final *R*_1_ was 0.0604 (I > 2σ(I)) and *wR*_2_ was 0.2024 (all data).

### 4.4. Computational Details

The ground state (S_0_) and the first singlet excited state (S_1_) geometries were optimized at the CAM-B3LYP/def2-SVP level using density functional theory (DFT) and time-dependent density functional theory (TD-DFT). Frequency analysis was performed to confirm the absence of imaginary frequencies, and the D3BJ dispersion correction was included throughout the calculations. Geometry optimizations were carried out using Gaussian 09 [25]. The reorganization energies for the S_1_ → S_0_ transition were decomposed with the DUSHIN code [26]. Molecular orbital distributions and electron–hole analyses were performed and visualized using Multiwfn (3.8 dev) and VMD software (version 1.9.3) [24,27,28,29].

## Data Availability

The data presented in this investigation is available from the corresponding authors.

## Supplementary Materials

The following supporting information can be downloaded at: https://www.mdpi.com/article/10.3390/molecules30214228/s1, Figure S1: UV-Vis absorption (red line) and photoluminescence (blue line) spectra of the compounds (a) PPA-3F and (b) PP-6F in CH_2_Cl_2_. The inserted numbers indicate the emission full width at half maximum (FWHM) and the Stokes shift. For PPA-3F, λₑₓ = 370 nm; for PP-6F, λₑₓ = 300 nm; Scheme S1: Molecular structures of the unmodified PP derivatives PP-8 and PP-9, along with their absorption and emission wavelengths in dichloromethane; Figure S2: Emission (a,c) and absorption (b,d) spectra of PPA-3F (a,b) and PP-6F (c,d) measured in solvents of varying polarity. For PPA-3F, λₑₓ = 370 nm; for PP-6F, λₑₓ = 300 nm; Figure S3: Steady-state photoluminescence (red line) and phosphorescence (blue line) spectra of compounds (a) PPA-3F (λ_ex_ = 370 nm) and (b) PP-6F (λ_ex_ = 340 nm) in toluene at 77 K. Steady-state emission spectra of compounds (c) PPA-3F and (d) PP-6F in toluene under air, nitrogen, and oxygen atmospheres (λ_ex_ = 300 nm); Figure S4: Photoluminescence decay curves of (a,c) PPA-3F and (b,d) PP-6F in dichloromethane (a–b) and in PS films (c–d). The monitoring wavelengths are 517 nm for (a), 402 nm for (b), 496 nm for (c), and 406 nm for (d); Figure S5: Emission spectra of PPA-3F (red lines) and PP-6F (blue lines) under ultraviolet excitation (solid lines) and X-ray excitation (dashed lines). For PPA-3F, λₑₓ = 370 nm; for PP-6F, λₑₓ = 300 nm; Figure S6: Photoluminescence spectra of compounds PPA-3F and PP-6F doped in polystyrene (PS) films at a concentration of 1 wt%. (λ_ex_ = 300 nm); Figure S7: Photoluminescence spectra of compounds PPA-3F and PP-6F in the solid (powder) state. (λ_ex_ = 370 nm); Figure S8: Singlet and triplet excited-state energy levels calculated based on the S_1_-optimized structures for (a) PPA-3F and (b) PP-6F; Table S1: Crystallographic data for PPA-3F and PP-6A; Figure S9: The ^1^H NMR spectra of PPA-3F; Figure S10: The ^13^C NMR spectra of PPA-3F; Figure S11: The ^1^H NMR spectra of PP-6F; Figure S12: The ^13^C NMR spectra of PP-6F; Figure S13: MS spectra of PPA-3F; Figure S14: MS spectra of PP-6F.

## References

[1] Wu X., Ni S., Wang C.-H., Zhu W., Chou P.-T. (2025). Comprehensive Review on the Structural Diversity and Versatility of Multi-Resonance Fluorescence Emitters: Advance, Challenges, and Prospects toward OLEDs. Chem. Rev..

[2] Mamada M., Hayakawa M., Ochi J., Hatakeyama T. (2024). Organoboron-Based Multiple-Resonance Emitters: Synthesis, Structure–Property Correlations, and Prospects. Chem. Soc. Rev..

[3] Hoppe H., Sariciftci N.S. (2004). Organic Solar Cells: An Overview. J. Mater. Res..

[4] Yi J., Zhang G., Yu H., Yan H. (2024). Advantages, Challenges and Molecular Design of Different Material Types Used in Organic Solar Cells. Nat. Rev. Mater..

[5] Liang W., Chen L., Wang Z., Peng Z., Zhu L., Kwok C.H., Yu H., Xiong W., Li T., Zhang Z. (2024). Oligothiophene Additive-Assisted Morphology Control and Recombination Suppression Enable High-Performance Organic Solar Cells. Adv. Energy Mater..

[6] Li J., Xie D., Yuan X., Li Y., Wei W., Zhang Y., Feng H., Luo X., Zhu J., Qin Z. (2025). Nucleation Driving Force-Controlled Fibril Network Formation Using a Non-Halogenated Solvent Enables Polythiophene Solar Cells with over 18% Efficiency. Energy Environ. Sci..

[7] Zhou Y., Zhang K., Chen Z., Zhang H. (2023). Molecular Design Concept for Enhancement Charge Carrier Mobility in OFETs: A Review. Materials.

[8] Fan X., Hao X., Huang F., Yu J., Wang K., Zhang X. (2023). RGB Thermally Activated Delayed Fluorescence Emitters for Organic Light-Emitting Diodes toward Realizing the BT.2020 Standard. Adv. Sci..

[9] Huang Y., Wang Z., Chen Z., Zhang Q. (2019). Organic Cocrystals: Beyond Electrical Conductivities and Field-Effect Transistors (FETs). Angew. Chem. Int. Ed..

[10] Stecko S., Gryko D.T. (2022). Multifunctional Heteropentalenes: From Synthesis to Optoelectronic Applications. JACS Au.

[11] Górski K., Shelton S., Lingagouder J., Data P., Jacquemin D., Gryko D.T. (2025). 1,4-Dihydropyrrolo[3,2- *b* ]Pyrrole Modified with Dibenzoxazepine: A Highly Efficient Core for Charge-Transfer-Based OLED Emitters. Chem. Sci..

[12] Szymański B., Sahoo S.R., Vakuliuk O., Valiev R., Ramazanov R., Łaski P., Jarzembska K.N., Kamiński R., Teimouri M.B., Baryshnikov G. (2025). Shedding New Light on Quadrupolar 1,4-Dihydropyrrolo[3,2- *b* ]Pyrroles: Impact of Electron-Deficient Scaffolds over Emission. Chem. Sci..

[13] Govind C., Balanikas E., Sanil G., Gryko D.T., Vauthey E. (2024). Structural and Solvent Modulation of Symmetry-Breaking Charge-Transfer Pathways in Molecular Triads. Chem. Sci..

[14] Krzeszewski M., Thorsted B., Brewer J., Gryko D.T. (2014). Tetraaryl-, Pentaaryl-, and Hexaaryl-1,4-Dihydropyrrolo[3,2-b]Pyrroles: Synthesis and Optical Properties. J. Org. Chem..

[15] Tasior M., Vakuliuk O., Koga D., Koszarna B., Gorski K., Grzybowski M., Kielesinski L., Krzeszewski M., Gryko D.T. (2020). Method for the Large-Scale Synthesis of Multifunctional 1,4-Dihydro-Pyrrolo[3,2-b]Pyrroles. J. Org. Chem..

[16] Krzeszewski M., Gryko D.T. (2015). Chi-Shaped Bis(Areno)-1,4-Dihydropyrrolo[3,2-b]Pyrroles Generated by Oxidative Aromatic Coupling. J. Org. Chem..

[17] Uoyama H., Goushi K., Shizu K., Nomura H., Adachi C. (2012). Highly Efficient Organic Light-Emitting Diodes from Delayed Fluorescence. Nature.

[18] Su J., Wei J., Ye K., Li F., Wang M., Li Q., Yuan A., Zhao Q., Shi C. (2025). Tuning Charge Transfer Properties in Symmetric and Asymmetric Pyrrolo[3,2- *b* ]Pyrrole Derivatives with Hybridized Local and Charge-Transfer Characteristics. Chem. Commun..

[19] Wang X., Shi H., Ma H., Ye W., Song L., Zan J., Yao X., Ou X., Yang G., Zhao Z. (2021). Organic Phosphors with Bright Triplet Excitons for Efficient X-ray-Excited Luminescence. Nat. Photonics.

[20] Dong M., Wang Z., Lin Z., Zhang Y., Chen Z., Wu Y., Ma H., An Z., Gu L., Huang W. (2025). Temperature-Adaptive Organic Scintillators for X-Ray Radiography. J. Am. Chem. Soc..

[21] Zhou Z., Wang X., Lv A., Ding M., Song Z., Ma H., An Z., Huang W. (2024). Achieving Efficient X-ray Scintillation of Purely Organic Phosphorescent Materials by Chromophore Confinement. Adv. Mater..

[22] Luo X.-F., Xiao X., Zheng Y.-X. (2024). Recent Progress in Multi-Resonance Thermally Activated Delayed Fluorescence Emitters with an Efficient Reverse Intersystem Crossing Process. Chem. Commun..

[23] Janiga A., Glodkowska-Mrowka E., Stoklosa T., Gryko D.T. (2013). Synthesis and Optical Properties of Tetraaryl-1,4-dihydropyrrolo[3,2-*b* ]Pyrroles. Asian J. Org. Chem..

[24] Liu Z., Lu T., Chen Q. (2020). An Sp-Hybridized All-Carboatomic Ring, Cyclo[18]Carbon: Electronic Structure, Electronic Spectrum, and Optical Nonlinearity. Carbon.

[25] Frisch M.J., Trucks G.W., Schlegel H.B., Scuseria G.E., Robb M.A., Cheeseman J.R., Scalmani G., Barone V., Mennucci B., Petersson G.A. (2016). Gaussian 09, Revision E.01.

[26] Reimers J.R. (2001). A Practical Method for the Use of Curvilinear Coordinates in Calculations of Normal-Mode-Projected Displacements and Duschinsky Rotation Matrices for Large Molecules. J. Chem. Phys..

[27] Lu T., Chen F. (2012). Multiwfn: A Multifunctional Wavefunction Analyzer. J. Comput. Chem..

[28] Humphrey W., Dalke A., Schulten K. (1996). VMD: Visual Molecular Dynamics. J. Mol. Graph..

[29] Lu T. (2024). A Comprehensive Electron Wavefunction Analysis Toolbox for Chemists, Multiwfn. J. Chem. Phys..

