# Supplementary material for: Tuning Excited-State Properties in Pyrrolo[3,2-b]pyrrole-Based Donor–Acceptor Emitters via Molecular Conformation and Conjugation Control
Source: Molecules. 2025 Oct 29;30(21):4228. doi: 10.3390/molecules30214228 (PMC12609304; doi:10.3390/molecules30214228)
Supplement: Supplementary file 1 [file molecules-30-04228-s001.zip › molecules-3954949-supplementary.pdf]

Supporting Information

Tuning Excited-State Properties in Pyrrolo[3,2-*b*]pyrrole-Based Donor–Acceptor Emitters via Molecular Conformation and Conjugation Control

Taotao Gan [1], Jie Su [1], Feiyang Li [1,], Qiuxia Li [1] and Chao Shi [1,*]*

[1] School of Environmental and Chemical Engineering, Jiangsu University of Science and Technology, Zhenjiang 212100, China. E-mail: lifeiyang@just.edu.cn, shichao@just.edu.cn.

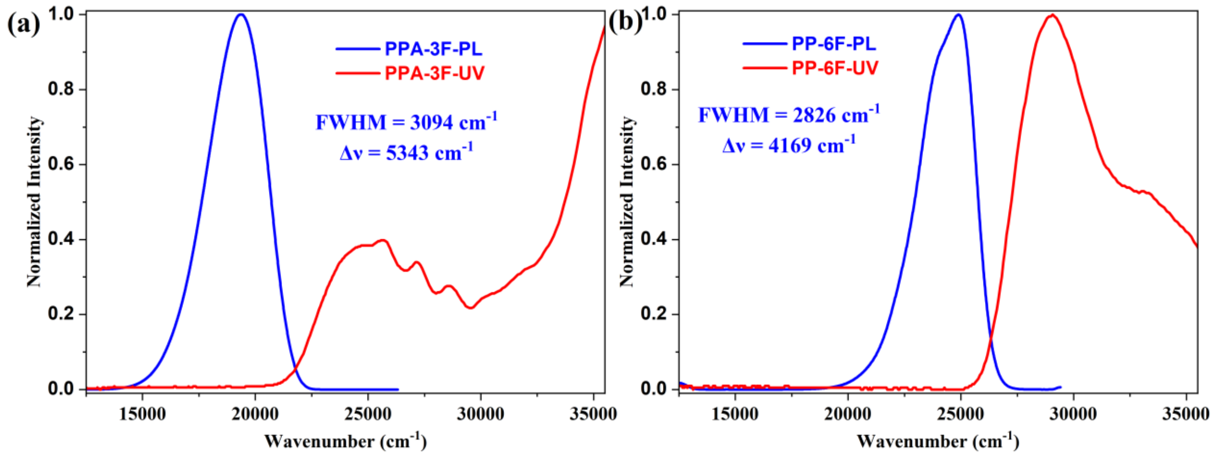

Figure S1. UV-Vis absorption (red line) and photoluminescence (blue line) spectra of the compounds (a) PPA-3F and (b) PP-6F in CH_2Cl_2. The inserted numbers indicate the emission full width at half maximum (FWHM) and the Stokes shift. For PPA-3F, λ_{ex} = 370 nm; for PP-6F, λ_{ex} = 300 nm.

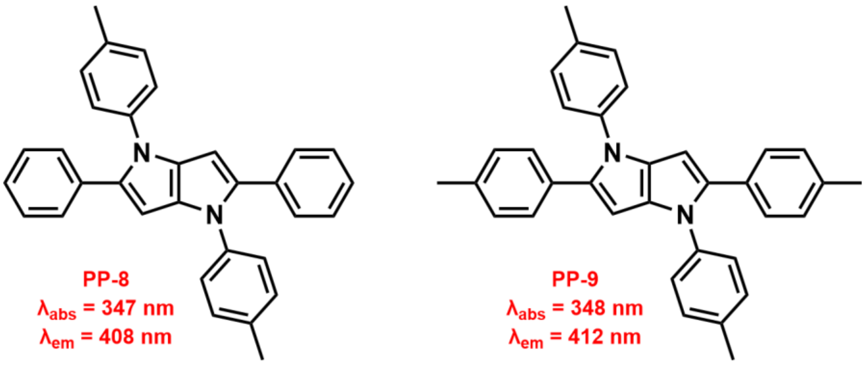

Scheme S1. Molecular structures of the unmodified PP derivatives PP-8 and PP-9, along with their absorption and emission wavelengths in dichloromethane.

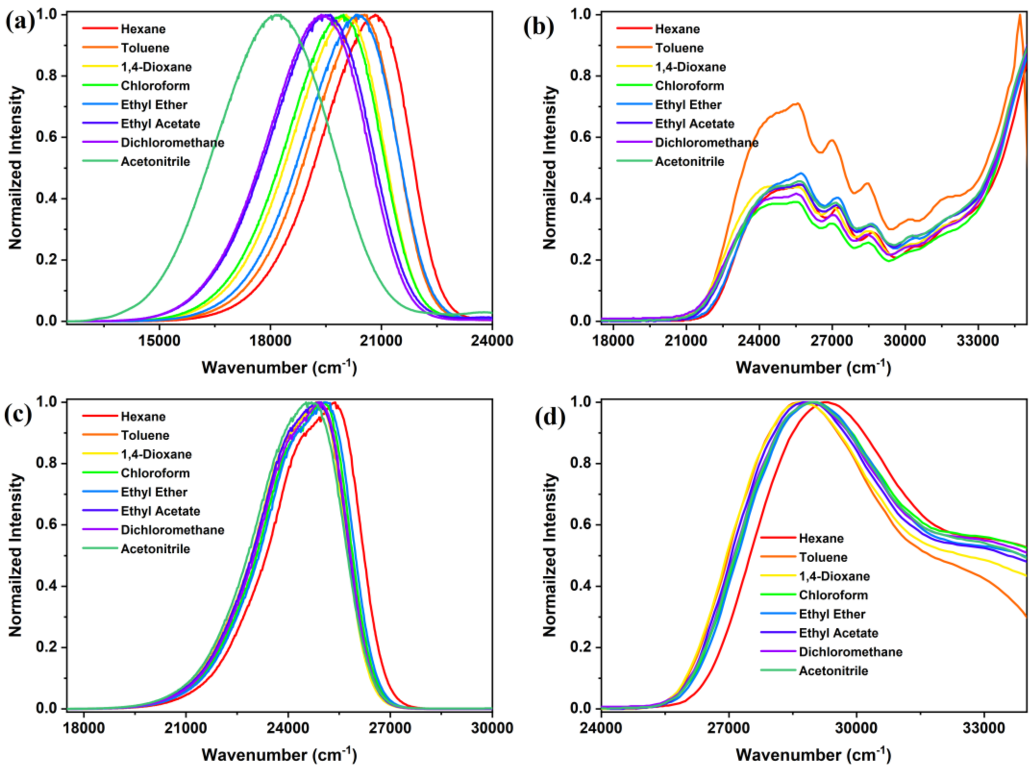

Figure S2. Emission (a, c) and absorption (b, d) spectra of PPA-3F (a, b) and PP-6F (c, d) measured in solvents of varying polarity. For PPA-3F, λ_{ex} = 370 nm; for PP-6F, λ_{ex} = 300 nm.

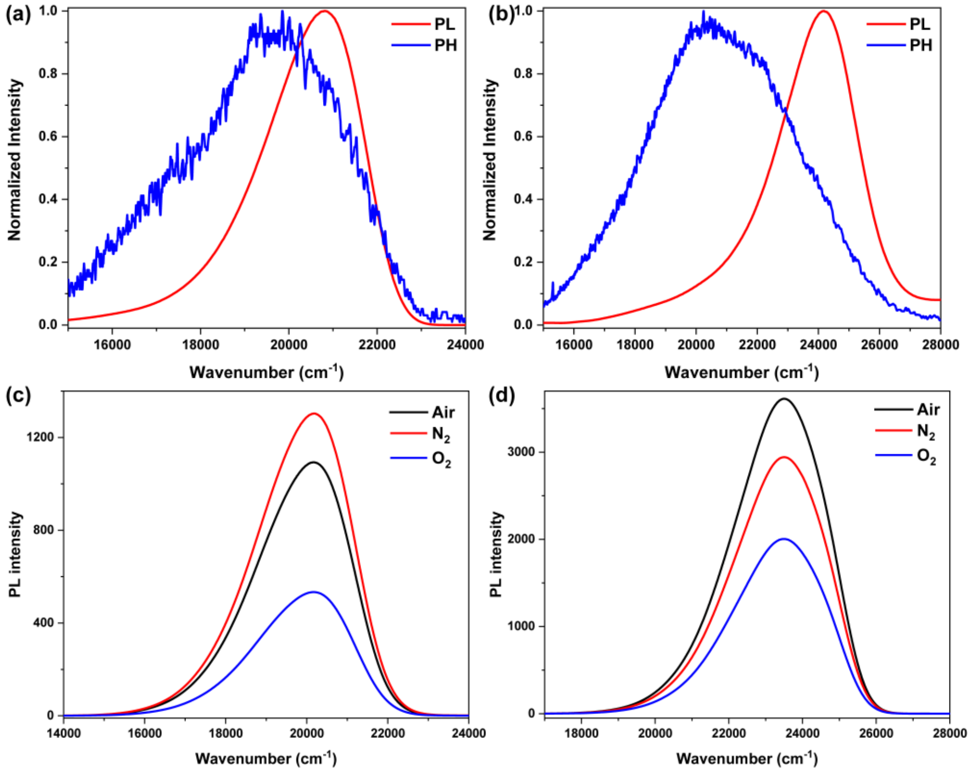

Figure S3. Steady-state photoluminescence (red line) and phosphorescence (blue line) spectra of compounds (a) PPA-3F (λ_{ex} = 370 nm) and (b) PP-6F (λ_{ex} = 340 nm) in toluene at 77 K. Steady-state emission spectra of compounds (c) PPA-3F and (d) PP-6F in toluene under air, nitrogen, and oxygen atmospheres (λ_{ex} = 300 nm).

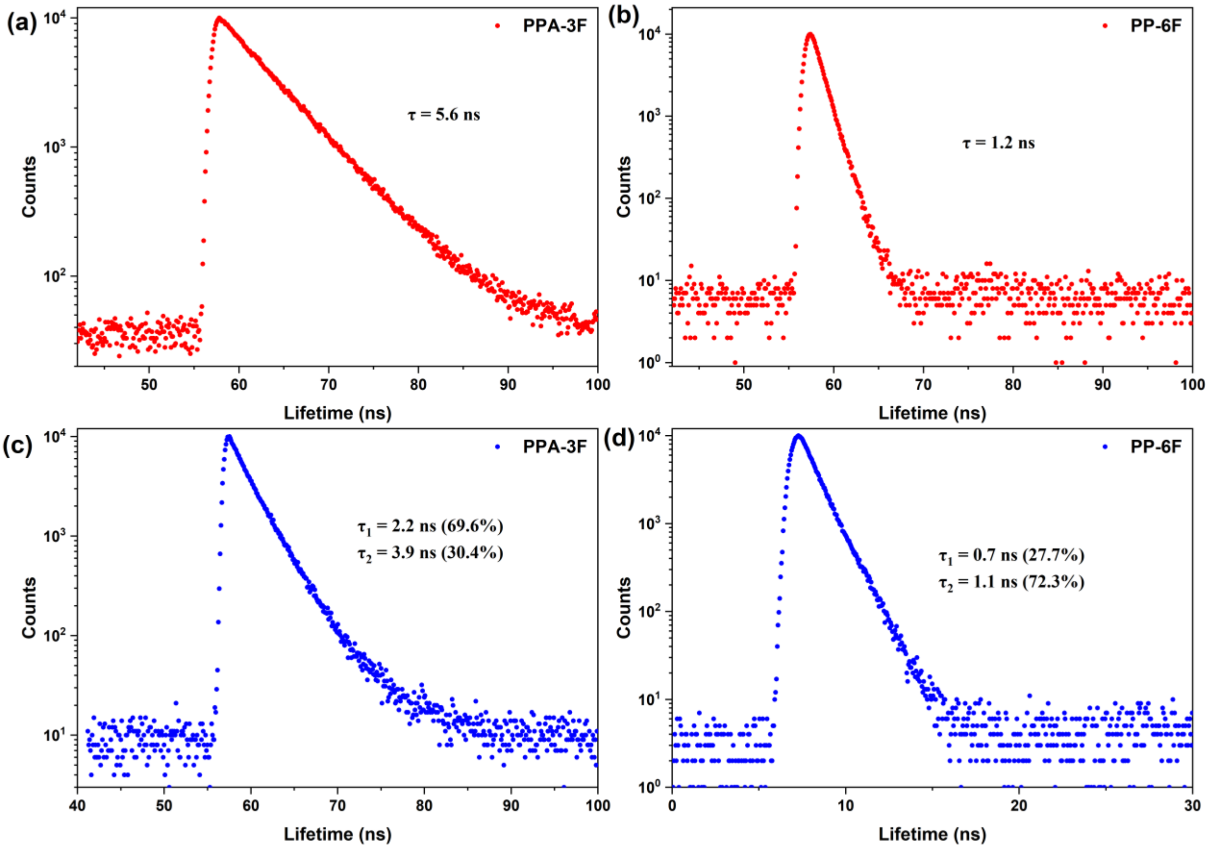

Figure S4. Photoluminescence decay curves of (a, c) PPA-3F and (b, d) PP-6F in dichloromethane (a–b) and in PS films (c–d). The monitoring wavelengths are 517 nm for (a), 402 nm for (b), 496 nm for (c), and 406 nm for (d). λ_{ex} = 360 nm

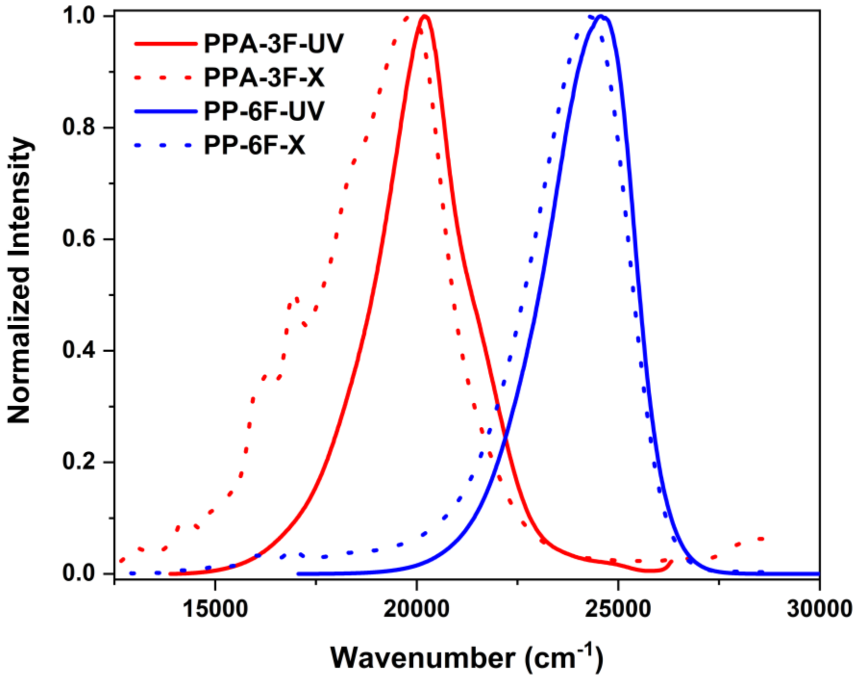

Figure S5. Emission spectra of PPA-3F (red lines) and PP-6F (blue lines) under ultraviolet excitation (solid lines) and X-ray excitation (dashed lines). For PPA-3F, λ_{ex} = 370 nm; for PP-6F, λ_{ex} = 300 nm.

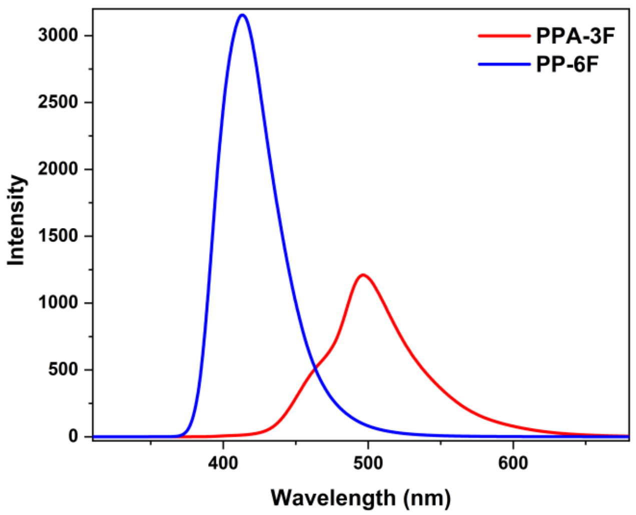

Figure S6. Photoluminescence spectra of compounds PPA-3F and PP-6F doped in polystyrene (PS) films at a concentration of 1 wt%. (λ_{ex} = 300 nm)

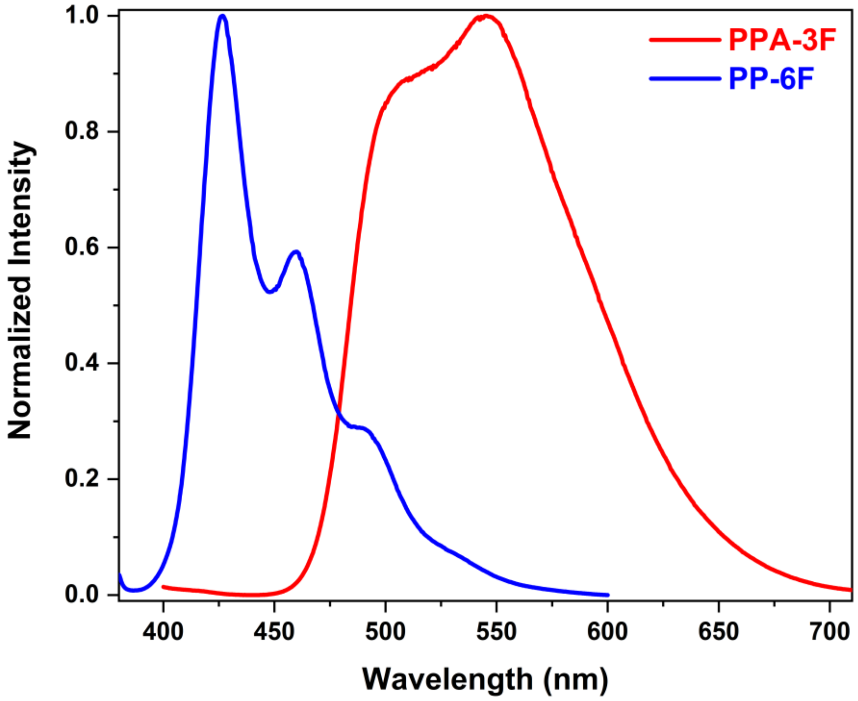

Figure S7. Photoluminescence spectra of compounds PPA-3F and PP-6F in the solid (powder) state. (λ_{ex} = 370 nm)

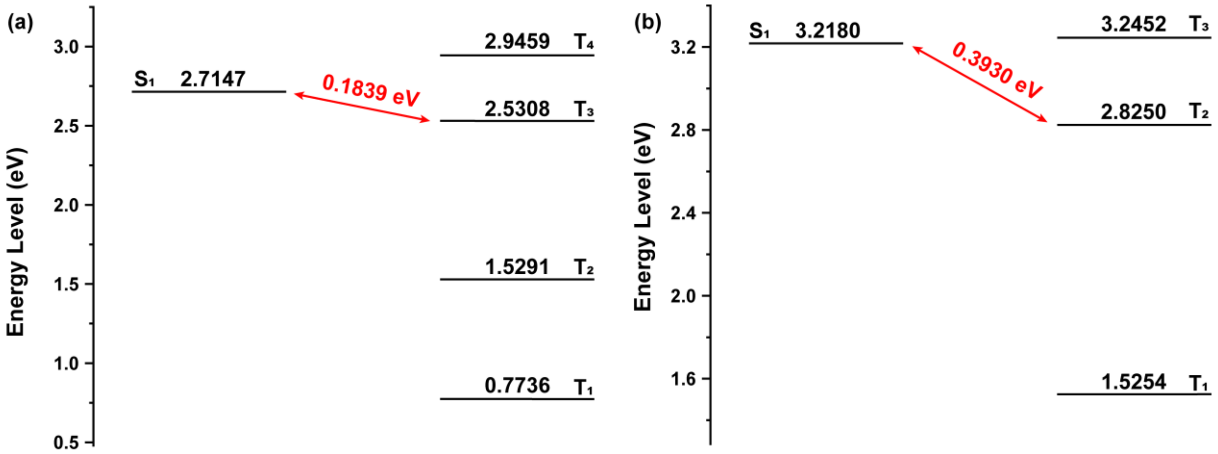

Figure S8. Singlet and triplet excited-state energy levels calculated based on the S_1-optimized structures for (a) PPA-3F and (b) PP-6F.

Table S1 Crystallographic data for PPA-3F and PP-6A

Identification code	PPA-3F	PP-6A
Empirical formula	$C_{46}H_{24}F_6N_2$	$C_{30}H_{10}F_{12}N_2$
Formula weight	718.67	626.4
Temperature/K	293.15	302
Crystal system	monoclinic	triclinic
Space group	$P2_1/n$	P-1
a/Å	8.732(4)	11.4197(9)
b/Å	14.019(5)	11.7546(10)
c/Å	13.610(6)	11.8294(9)
α/°	90	111.681(4)
β/°	97.103(7)	99.552(4)
γ/°	90	102.922(4)
Volume/Å^3	1653.2(11)	1383.0(2)
Z	2	2
ρ_{calc}g/cm^3	1.444	1.504
μ/mm^{-1}	0.108	1.305
F(000)	736	624
Radiation	MoKα (λ = 0.71073)	CuKα (λ = 1.54178)
2Θ range for data collection/°	5.526 to 56.658	8.26 to 136.732
Index ranges	$-11 \le h \le 9, -17 \le k \le 18, -18 \le l \le 18$	$-13 \le h \le 13, -14 \le k \le 13, -14 \le l \le 11$
Reflections collected	14852	15308
Independent reflections	4121 [R_{int} = 0.0373, R_{sigma} = 0.0395]	5014 [R_{int} = 0.0497, R_{sigma} = 0.0621]
Data/restraints/parameters	4121/0/244	5014/0/397
Goodness-of-fit on F^2	1.023	1.125
Final R indexes [I>=2σ (I)]	R_1 = 0.0468, wR_2 = 0.0959	R_1 = 0.0604, wR_2 = 0.1914
Final R indexes [all data]	R_1 = 0.0943, wR_2 = 0.1179	R_1 = 0.0904, wR_2 = 0.2024
Largest diff. peak/hole / e Å^{-3}	0.16/-0.19	0.30/-0.24

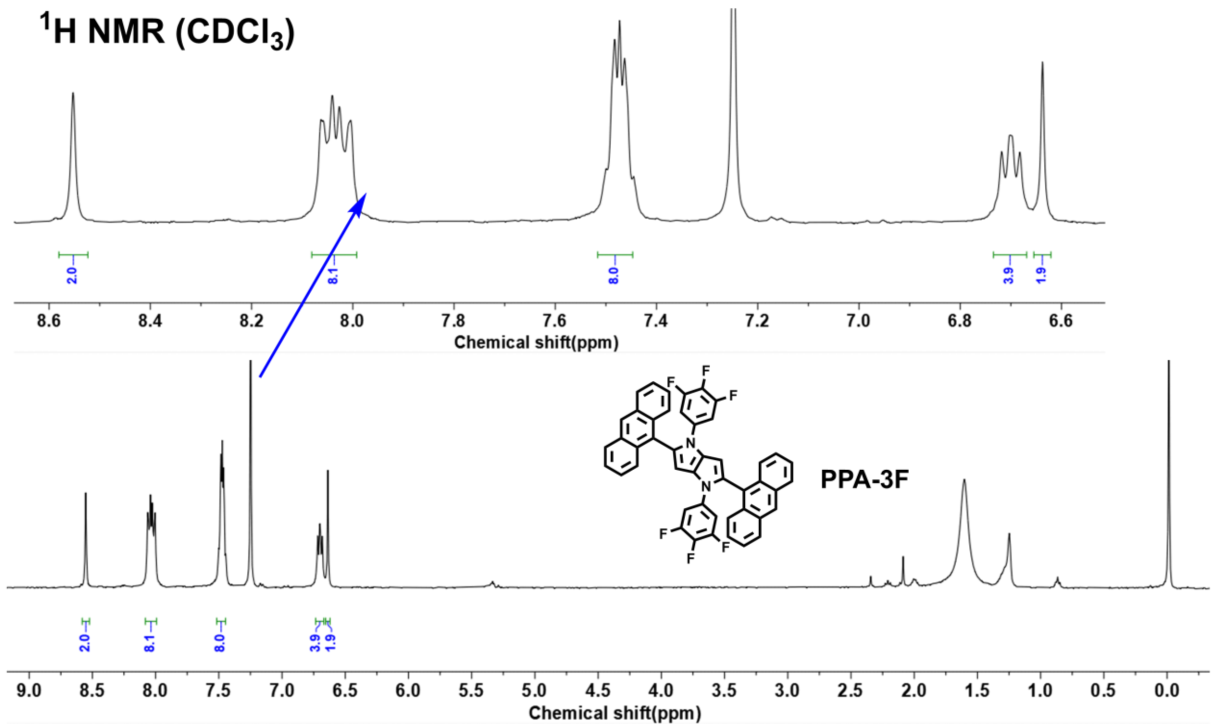

Figure S9. The ^{1}H NMR spectra of **PPA-3F**.

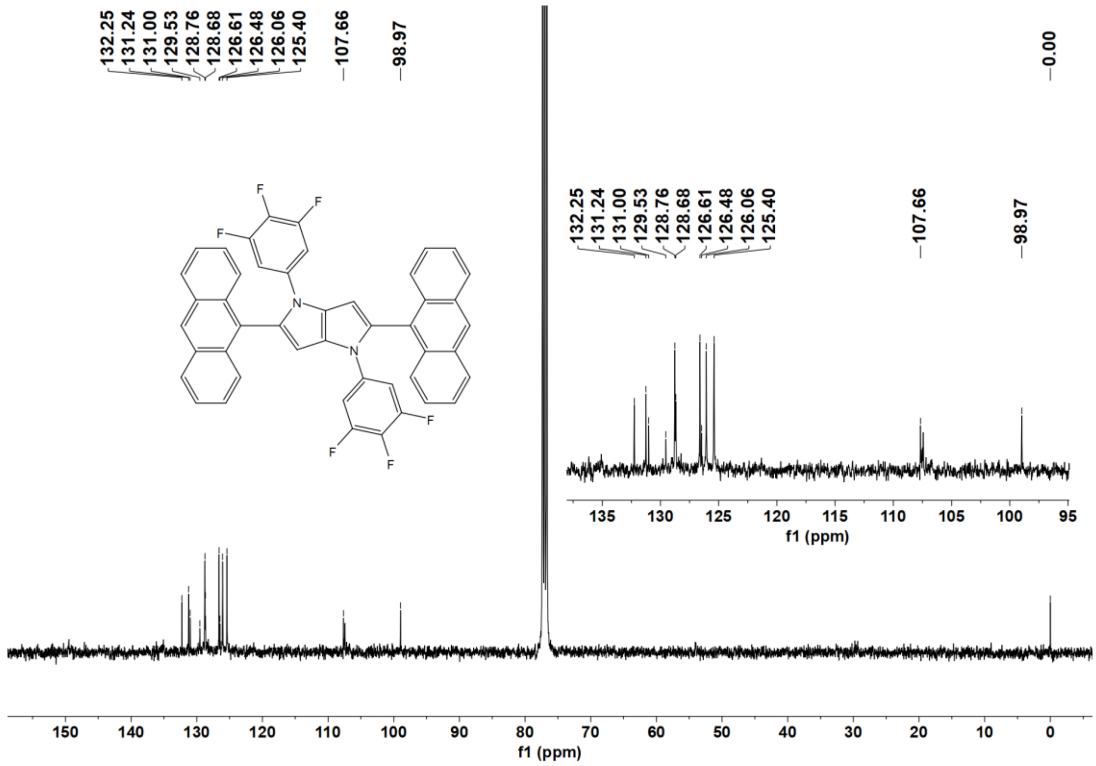

Figure S10. The ^{13}C NMR spectra of **PPA-3F**.

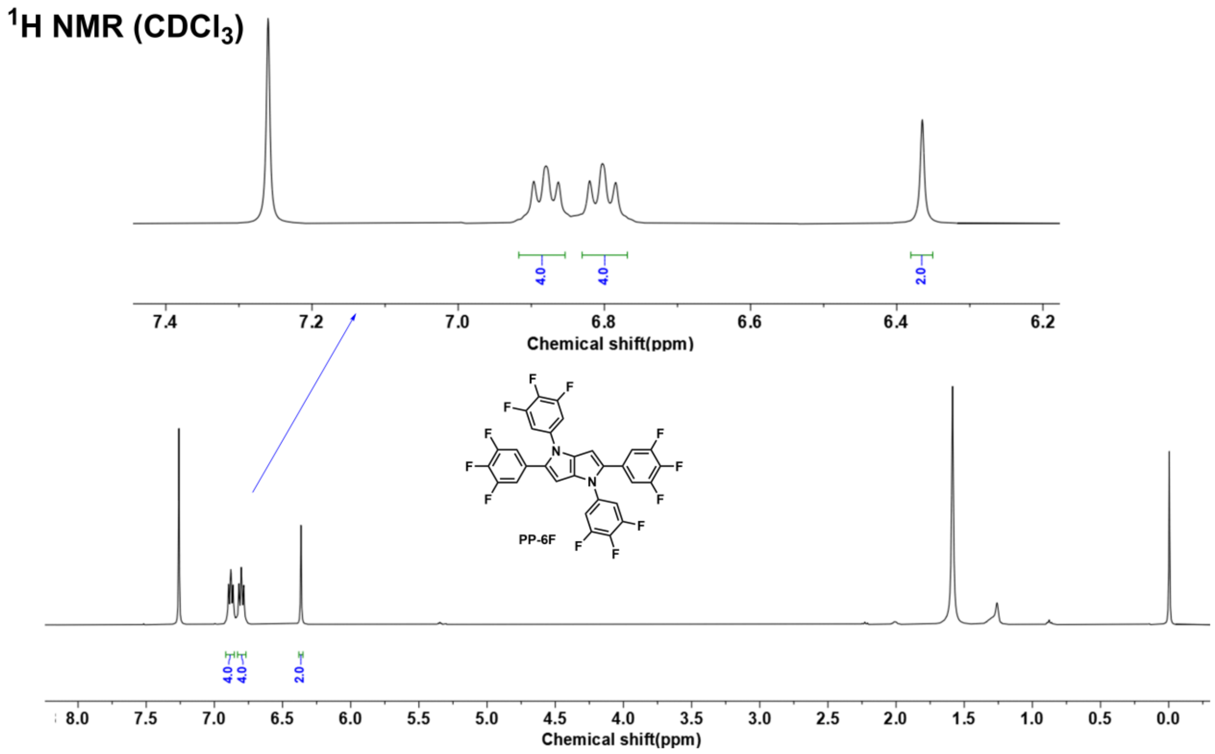

Figure S11. The ^{1}H NMR spectra of **PP-6F**.

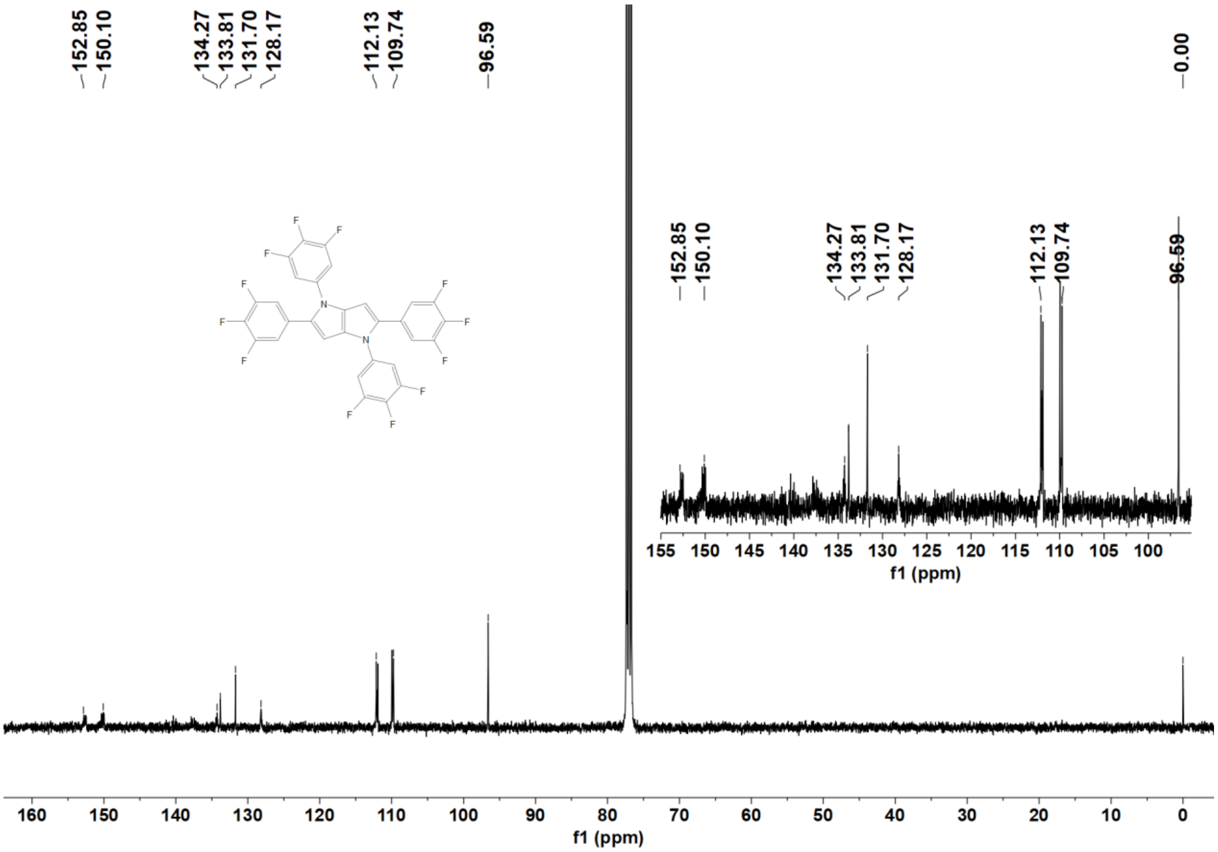

Figure S12. The ^{13}C NMR spectra of **PP-6F**.

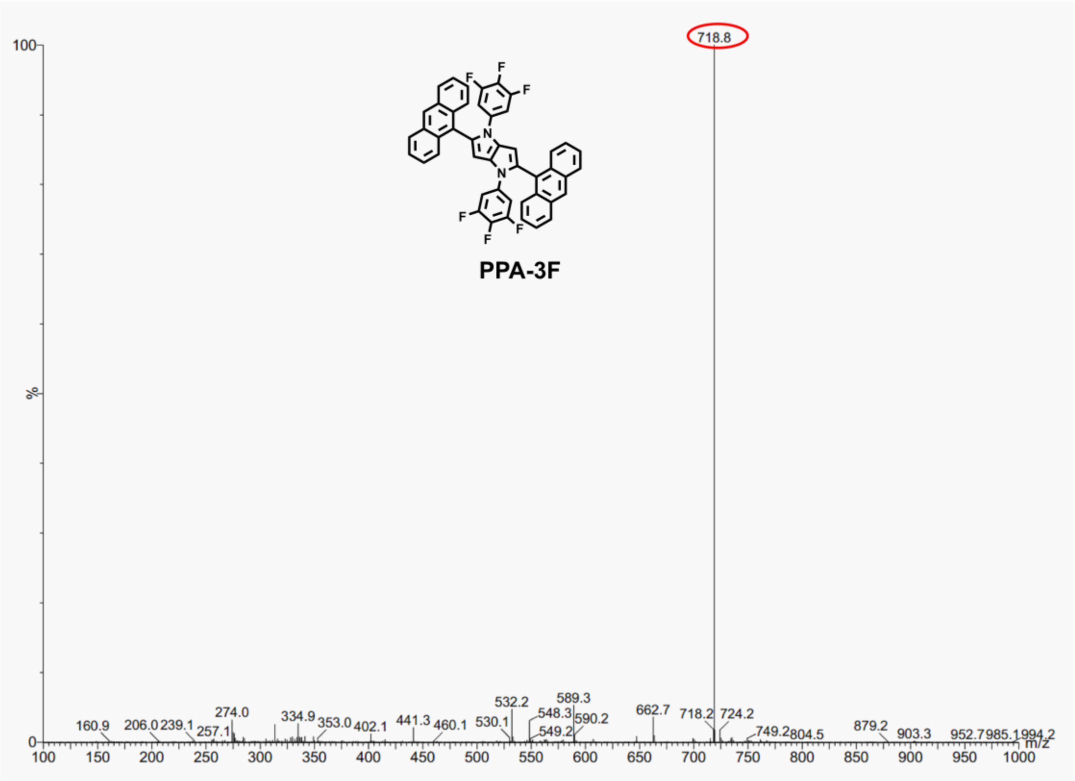

Figure S13. MS spectra of **PPA-3F**.

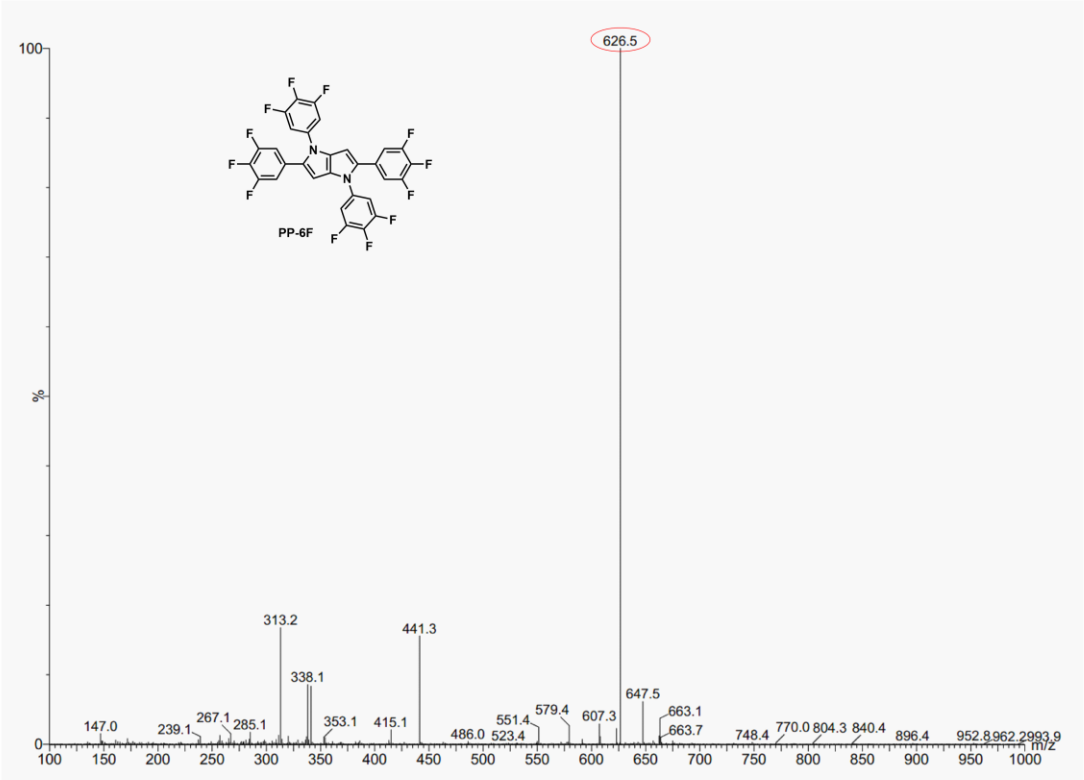

Figure S14. MS spectra of **PP-6F**
